# Microcin C7 as a Potential Antibacterial-Immunomodulatory Agent in the Postantibiotic Era: Overview of Its Bioactivity Aspects and Applications

**DOI:** 10.3390/ijms25137213

**Published:** 2024-06-29

**Authors:** Fengjuan Yang, Feiyun Yang, Jinxiu Huang, Haitao Yu, Shiyan Qiao

**Affiliations:** 1 State Key Laboratory of Animal Nutrition and Feeding, Ministry of Agriculture and Rural Affairs Feed Industry Centre, China Agricultural University, Beijing 100193, China; yangfengjuan@cau.edu.cn (F.Y.); qiaoshiyan@cau.edu.cn (S.Q.); 2 Beijing Biofeed Additives Key Laboratory, Beijing 100193, China; 3 Chongqing Academy of Animal Science, Rongchang, Chongqing 402460, China; yfeiyun@yeah.net (F.Y.); short00@163.com (J.H.); 4 National Center of Technology Innovation for Pigs, Rongchang, Chongqing 402460, China

**Keywords:** Microcin C7, antibacterial and immunoregulatory effects, bioactivity aspects, animal applications

## Abstract

In the postantibiotic era, the pathogenicity and resistance of pathogens have increased, leading to an increase in intestinal inflammatory disease. Bacterial infections remain the leading cause of animal mortality. With increasing resistance to antibiotics, there has been a significant decrease in resistance to both inflammation and disease in animals, thus decreasing production efficiency and increasing production costs. These side effects have serious consequences and have detracted from the development of China’s pig industry. Microcin C7 (McC7) demonstrates potent antibacterial activity against a broad spectrum of pathogens, stable physicochemical properties, and low toxicity, reducing the likelihood of resistance development. Thus, McC7 has received increasing attention as a potential clinical antibacterial and immunomodulatory agent. McC7 has the potential to serve as a new generation of antibiotic substitutes; however, its commercial applications in the livestock and poultry industry have been limited. In this review, we summarize and discuss the biosynthesis, biochemical properties, structural characteristics, mechanism of action, and immune strategies of McC7. We also describe the ability of McC7 to improve intestinal health. Our aim in this study was to provide a theoretical basis for the application of McC7 as a new feed additive or new veterinary drug in the livestock and poultry breeding industry, thus providing a new strategy for alleviating resistance through feed and mitigating drug resistance. Furthermore, this review provides insight into the new functions and anti-infection mechanisms of bacteriocin peptides and proposes crucial ideas for the research, product development, and application of bacteriocin peptides in different fields, such as the food and medical industries.

## 1. Introduction

Bacterial resistance remains one of the most serious global public health issues in the postantibiotic era. The pathogenicity and resistance of pathogens have been intensifying, and subsequent infections have caused significant public health problems and incurred immense medical and financial costs [1,2,3,4]. In addition, a variety of diseases, such as intestinal inflammatory disease (IBD), chronic pneumonia, bacterial liver abscess, bacillary dysentery, and sepsis, are caused by bacterial infections and are characterized by being easy to infect, easy to circulate, difficult to predict, and difficult to control [5,6]. Toxins or other metabolites produced by pathogens can lead to cell, tissue, and organ dysfunction or death [7]. For example, the intestinal digestive system and immune system in neonates and young animals are not yet fully understood, and infection with pathogenic microorganisms can lead to increased intestinal permeability, impaired barrier function, and a leaky gut. Ultimately, mucosal-associated gram-negative bacteria and lipopolysaccharide (LPS) via the leaky gut induce an imbalance in intestinal microbiota homeostasis and an excessive intestinal inflammatory response, as well as organ injury [8,9,10,11,12]. With increasing pathogen resistance, the efficacy of antibiotics in treating various bacterial infections has been affected. If the bacterial resistance problem is not solved and new antimicrobial agents are not discovered in time, bacterial infections could have extremely high morbidity and mortality. Thus, new strategies to address antibiotic resistance are urgently needed.

In the modern pig industry, the growth and health status of piglets during their neonatal and weaning periods are critical for their lifelong well-being and are key factors that influence production efficiency and profitability. Limitations on the use of antibiotics have provided limited relief to the bacterial resistance problem, but waning disease resistance and production efficiency and the associated increase in production costs have become prominent [13,14,15]. These challenges have significantly impacted the efficiency of the productive performance of domestic animals. Therefore, there is an urgent need for the sustainable development of animal husbandry through the development of feasible, efficient, natural, and safe antibiotic substitutes. Moreover, it is important to analyze the mechanism of action and solve the core scientific issues related to the use of antibiotic substitutes. In recent years, many alternatives to traditional antibiotics, such as probiotics, enzymes, micro- and nanobiomaterials, and antibacterial peptides (AMPs), have been extensively researched and used [16,17,18,19,20,21,22,23,24].

The anti-infective effects of AMPs on a wide range of pathogenic microorganisms are one of their important biological characteristics. Many studies have shown that the direct inhibition or killing of pathogens and the regulation of immune function are the two main ways that AMPs exert their anti-infective effects [25,26]. In addition, recent studies have shown that AMPs play an important role in intestinal homeostasis, for example, not only through relieving intestinal inflammation but also through improving intestinal barrier functions [10,12,27]. An in-depth understanding of the structure, function and anti-infection mechanisms of AMPs has promoted clinical research and the application of AMPs, and AMPs are now considered the most promising potential substitutes for traditional antibiotics [28,29,30,31,32]. Bacteriocins are an AMP subclass that exhibit strong antibacterial activity in treating target strains. In contrast to antibiotics, most bacteriocins are selective and only have antibacterial properties against species similar to their producer strains [33]. A few years ago, this narrow antibacterial spectrum was a major bottleneck in AMP application; however, we now know that the destruction of the gut microbiota caused by broad-spectrum antibiotics is associated with immunological, metabolic, and neurological diseases. Therefore, bacteriocins are also more efficient and less invasive to the microbiota through targeting specific bacteria. Among the alternatives to antibiotics used in traditional therapies, bacteriocins are considered the most promising antibacterial drugs for the future [34,35,36].

Microcins, plasmid-encoded, small antimicrobial peptides from the bacteriocin family synthesized in ribosomes by *Escherichia coli* (*E. coli*), are less than 10 kDa in size and have strong antibacterial activity and stability. Microcins have been extensively studied and used due to their stable biochemical properties, weak toxicity, resistance to drugs and immune efficacy [33,37,38]. Microcins can be used by host intestinal bacteria to alleviate *Salmonella* and *E. coli* in the intestine, as well as to relieve intestinal inflammation [36]. Microcin J25 (MccJ25) has anti-infection effects under physiological and inflammatory conditions, such as alleviating the host inflammatory response, enhancing intestinal barrier function, and improving the intestinal microbiota and metabolic ability, thereby improving the host’s intestinal health [12,18,39]. Therefore, microcins are considered to be a new generation of antibiotic substitutes with great potential; however, they have rarely been commercially used in the livestock and poultry industries.

Microcin C7 (McC7) is encoded by ribosomes and is often more amenable to engineering than classical antibiotics. Biologically active biosynthetic McC7 can be obtained through genetic engineering and has further applications in clinical practices, which has made this material more attractive from the perspective of the productive performance of domestic animals [40,41,42]. However, to some extent, scientific evaluation and data on the practical application of the biosynthetic material McC7 as a feed additive in animal production are lacking. In this review, we first introduce the general aspects of bacteriocins to clarify their importance and further elucidate the reasons for their efficiency and selectivity and decipher in detail their mechanisms of action and potential application. For example, we summarize the physicochemical properties, antibacterial activity, and in vitro and in vivo safety and drug resistance of McC7 and elucidate its mode of action. Based on its bioactivities, we further discuss its potential anti-infection function and potential for use in the poultry and swine industries as a promising alternative to conventional antibiotics. Finally, we summarize and discuss promising strategies to improve the antibacterial ability of McC7 and provide potential research directions (for pathogenic infections and anti-inflammatory diseases) for interested readers.

## 2. Bacteriocins and Their Classifications

AMPs are produced throughout the entire biological realm, from animals and plants to microorganisms. In recent years, with increasing antibiotic resistance, it has been found that AMPs secreted by bacteria typically have more antibacterial effects than those produced by eukaryotes [43]. Bacteriocins are peptides synthesized in ribosomes that are directly encoded by genes; these peptides have the advantages of low toxicity and high-titer bacteriocins and are often more amenable to engineering than classical antibiotics [33]. Different bacteriocins have various structures and mechanisms of action and possess a narrow range of activity toward their own specific microbial targets [44]. They exert especially strong activity only against closely related species and, thus, are considered the most promising new generation of antimicrobial agents. Bacteriocins are ubiquitous in nature. In theory, each microorganism can produce at least one antimicrobial protein under specific conditions, such as nutrient depletion or fierce niche competition [45]. Many microorganisms do not show antibacterial activity against other microorganisms due to limitations in production conditions and/or monitoring technology [46]. With the continuous discovery and identification of new bacteriocins, such as those produced by many gram-positive bacteria, it is gradually being realized that they may constitute a unique class of compounds. The early criteria used to define bacteriocins were (1) narrow-spectrum antibacterial properties against similar species; (2) a biologically active component consisting of a peptide; (3) bactericidal activity; (4) binding to specific bacterial receptors; (5) encoded by a plasmid; and (6) death of the bacteria that produce it [45]. However, with an in-depth understanding of the synthesis, structure, and mechanism of bacteriocins, the above criteria can no longer accurately describe bacteriocins. For instance, many bacteriocins have broad inhibition spectra, are not encoded by plasmids, and contain immune genes. Therefore, a more accurate description of the term “bacteriocin” is a modified or unmodified AMP produced by bacteria that are protected by the immune system [47]. According to the producing strains, bacteria can be divided into two categories: gram-positive bacteria and gram-negative bacteria. Each category is based on its structure, and they are categorized based on their function and mode of action. Additionally, bacteriocins can be divided into classes according to their producing strain, molecular weight, thermal stability, and presence of a posttranslational modification.

### 2.1. The Bacteriocins Produced by Gram-Positive Bacteria

The bacteriocins produced by lactic acid bacteria (LAB) are the earliest and most studied gram-positive bacteria. They can be used for food preservation, food safety, and human and veterinary drugs due to their food-grade characteristics [48]. Therefore, a variety of classification methods for gram-positive bacterial bacteriocins have been established. The LAB bacteriocin classification standard proposed by Klaenhammer et al. (1993) is widely accepted [49]. Based on this classification standard, gram-positive bacteria were divided into four categories, with each category further divided into subcategories.

The first category is posttranslationally modified bacteriocins, denoted as class I. Class I bacteriocins have a molecular weight < 5 kDa and are posttranslationally modified and thermostable lanthionine bacteriocins (Lantibotics), which are encoded by genes located on plasmids or chromosomes. They contain special amino acids, such as lanthionine, β-methyllanthionine, and anhydrolanthionine, and can be further divided into three classes: Ia, Ib, and Ic. Class Ia specifically refers to lantibiotics, with typical representatives being nisin, Pep5, and epidermin. Class Ib contains a posttranslational modification of the amino acid (AA) labin with the “Labyrinthine” structure; hence, it is named the Labyrinth protein. It inhibits herpes simplex virus and has shown potential in the treatment of neuropathic pain [50]. A representative class Ic peptide is subtilosin A, a cyclic peptide produced by *Bacillus* spp., which is different from all other class IId cyclic peptides (i.e., it has a lower molecular weight and is posttranslationally modified) [51]. Nisin is the most studied lanthionine bacteriocin. It inhibits cell wall synthesis through pore formation and specific binding to lipid II (the basic precursor to the bacterial cell wall), thereby exerting antibacterial activity as a food preservative [52]. Nisin also has antibacterial effects on a series of multidrug-resistant bacteria [53]. In addition, nisin is also used in the veterinary field, and a study has shown that it is active against gram-positive pathogens such as MRSA that cause bovine mastitis [54].

The second type is non-posttranslationally modified bacteriocins, denoted as class II. Class II bacteriocins are a type of peptide with a molecular weight less than 10 kDa but greater than 5 kDa. They are composed of standard AA. The molecules may contain disulfide bonds, or the N- and C-termini may be connected to form a loop [47]. The bacteriocins encoded by plasmids and without posttranslational modifications are called class IIa bacteriocins [55], while class IIb bacteriocins are linear bacteriocins with posttranslational modifications. They can exert antibacterial activity against some pathogenic bacteria, such as *Listeria monocytogenes,* at the nanomolar level and against some drug-resistant bacteria, such as methicillin-resistant *Staphylococcus aureus* (MRSA) and vancomycin-resistant *Enterococcus* (VRE) [44].

Class II bacteriocins comprise three groups: IIa, pediocin-like; IIb, two-component (with active bodies composed of two or more peptide chains); and IIc, sulfur-activated [49]. On this basis, Cotter et al. retained the two subclasses IIa and IIb and introduced classes IIc (cyclic peptides) and IId (nonpediocin single-chain peptides) [42]. This classification method has also been recognized by other researchers [56]. Class IIa bacteria can be produced by a variety of gram-positive bacteria, especially LAB, and exhibit strong antibacterial activity against *Listeria monocytogenes*, MRSA, and *Bacillus*. These bacteriocins are considered suitable for application in biomedicine and as natural food preservatives [47]. They are characterized by a narrow inhibition spectrum, but all can kill *Listeria monocytogenes,* with sizes ranging from 55 AA (acidocin A) to 37 AA (sakacin G) [57,58]. All the sequences contained the N-terminal conserved sequence YGNGV-X1-C-X2-K/N-X3-X4-C (where X is any AA). This hydrophilic and positively charged sequence has been named the “pediocin box”. Type IIb active entities are composed of two or more peptide chains, most of which are produced by LAB. The peptide chain residues are within 40 AA; for example, plantaricin S and lactocoscin G contain 26/24 and 39/35 residues, respectively [56,59]. For larger peptides such as brochocin-C, sakacin T, gassericin T, and lactacin F, at least one peptide chain is typically longer than 50 AA residues [60,61]. In all cases, the genes encoding these two bacteriocin components are arranged in tandem on the same operon, the immune proteins are encoded by adjacent genes, and the dedicated ABC transporter genes are also located on the same or adjacent operons. They have little or no activity in the separation process, and physical interaction is needed to form an active complex. Although the AA sequences and structures of class IIb bacteriocins vary, their peptide chains all contain the conserved GxxxG motif. In lactococcin G, the two peptide chains interact at the helix formed by the motifs to form an active complex [62]. Class IIc includes cyclic peptide bacteriocins, which are encoded by genes and synthesized by ribosomes. Posttranslational modification of precursor proteins leads to covalent linking of the N- and C-termini to form a circular backbone. The structures of carnocyclin A and AS-48 (two class IIc bacteriocins) were elucidated by nuclear magnetic resonance and X-ray diffraction. They contain a repetitive α-helical motif and are heat stable, resistant to proteolysis, and resistant to *Listeria* [63,64]. To date, eight class IIc bacteriocins have been discovered, among which six are produced by LAB (gassericin A, reutericin 6, enterocin AS-48, enterocin 4, carnocyclin A, and lactoin Q), and the other two are Cirularin A produced by *Clostridium bayeris* and butyrivibriocin AR10 produced by *Vibrio fibrinolyticus* [65,66,67,68,69]. They are subdivided into two subunits based on their amino acid (AA) sequence. Cotter et al. proposed two branches of IIc bacteriocins: IIc (i.), which contains enterocin AS-48 and ircularin A from non-LAB; and IIc (ii.), which includes gassericin A, reutericin 6, and AR10 produced by *Clostridium beijerinckii* [42]. In contrast, based on classification by the AA sequence, carnocyclin A, lactocyclin Q, AS-48, circularin A, and uberolysin were classified as class IIc (i.), while the highly homologous bacteriocins gassericin A, reutericin 6, and AR10 were classified as category IIc (ii.). [64]. Class IId peptides are unmodified, nonpediocin single-chain peptides. This group of bacteriocins contains antibacterial peptides from various strains in different niches, and lactococcin A was the first member of this class. Nissen-Meyer et al. classified 31 IId bacteriocins, mainly from LAB but also from *Staphylococcus* and *Bacillus* spp. For example, laterosporulin secreted by *Bacillus lateralis* GI-9 has a molecular weight of 5.6 kDa and has three disulfide bonds [56]. Additionally, laterosporulin is similar in structure to human defensins. Laterosporulin exhibits heat stability and protease resistance and can kill a variety of pathogenic microorganisms, including *E. coli*, *L. monocytogenes,* MRSA, *P. aeruginosa,* and *V. cholerae*. Due to these excellent biological functions, laterosporulin has potential medicinal value [70,71].

The third category includes large heat-sensitive proteins, which usually have enzymatic activity and are denoted as category III. They all contain different typical domains, including an ectopic domain, a receptor binding domain, and a lethal domain. Helveticin J is produced by *Lactobacillus helveticus* and contains five different domains [72]. The fourth type is a complex consisting of other molecules (lipids or carbohydrates) and proteins.

### 2.2. Most Gram-Negative Bacteriocins Are from Enterobacteriaceae

As the bacteriocins produced by gram-negative bacteria have unique structural motifs, they are separate from the bacteriocins produced by gram-positive bacteria, which have unique target organisms and diverse mechanisms. The bacteriocins secreted by gram-negative bacteria, mainly *E. coli* and other *Enterobacteriaceae*, are classified according to their molecular weight. Bacteriocins with a molecular weight of 1–10 kDa are called microcins, while those with a molecular weight of 30–80 kDa are called coliforms [73]. When bacterial DNA is damaged, the response to colicin is mediated by the SOS reaction, and the resulting plasmid contains structural genes, immune protein genes, and genes encoding lytic proteins. Microcins are highly stable hydrophobic peptides that are generally produced during cell growth to the stationary stage or under stress conditions, especially nutrient depletion [74]. Most colicins and microcins are produced by *E. coli*, but some other gram-negative bacteria can also produce similar substances, such as lumicins produced by the insect pathogenic bacterium *Photorhabdus luminescens* [75].

Colicins are produced in *E. coli* and are generally encoded by plasmids. They are usually coexpressed with lysis proteins; therefore, they are lethal to production strains. A typical colicin operon contains three types of genes: encoding genes, such as the encoding gene *cxa* of colicin X; immunity genes, which are generally located downstream of the encoding genes, such as the colicin X immunity gene *cxi*; and lysis proteins, which lyse bacteria to release colicins [76]. According to their bactericidal mechanism and uptake methods, colicins can be divided into Group A, Group B, and an undetermined group [73]. In fact, colicins have three bactericidal mechanisms [73,74]: (1) the formation of voltage-dependent channels on the intima of sensitive strains; (2) cytoplasmic degradation; and (3) peptidoglycan degradation. The intake of Group A requires the Tol protein, or one or more of these proteins; for example, the intake of colicin E1 requires TolA/TolQ, the intake of colicin N requires TolA/TolQ/TolR, and the intake of all Group B requires TonB/ExbB/ExbD proteins [77]. In addition, Group A is encoded by small plasmids and released into media (e.g., colicins A, E1-E9, K, N, U, and S4), while Group B is encoded by large plasmids and cannot be released into media (e.g., colicins B, D, Ia, and M) [73,78]. Most colicins contain three functional domains, and each domain corresponds to a step in the mode of action: (1) the central domain is involved in receptor binding, thereby recognizing and adhering to a specific region on the surface of target bacteria; (2) the N-terminal domain participates in transport, allowing colicins to enter target bacteria; and (3) the C-terminal domain is the active region that can kill target bacteria [73].

The second category consists of low-molecular-weight peptides called microcins. Microcins are bacteriocins with molecular weights of 1–10 kD produced by gram-negative bacteria (mainly *E. coli*). As a class of ribosomal synthetic peptides with a molecular weight less than 10 kDa, microcins have a dedicated export system for noninduced production and resistance to pH fluctuations, heat, and acidic and alkaline environments. Duquesne et al. divided microcins into two types, I and II, based on the following three principles: (1) the nature and positioning of posttranslational modifications; (2) the organization of the gene clusters; and (3) the sequence of the leader peptide [33,37]. The molecular weight of class I microcins is less than 5 kDa, and they include posttranslationally modified peptides, such as MccB17, McC7-C51, and MccJ25 [79]. Class II microcins have a molecular weight of 5–10 kDa and are further divided into two subclasses, IIa and IIb. Class IIa is encoded by a plasmid and has no posttranslational modification or disulfide bond formation (e.g., MccL, MccV, and Mcc24), while class IIb is encoded by a chromosomal gene with a posttranslational modification at the C-terminus (e.g., E492, M, and H47) [80].

In contrast to colicin, microcins are usually produced when cells reach the stationary phase or are under cell stress conditions (e.g., nutrient deficiency) [74]. Microcins are encoded by genes located on plasmids. The types of microcins are diverse, and most are unrelated to each other (Table 1). Three criteria are involved in their classification: (1) the existence, nature, and location of posttranslational modifications; (2) the presence of gene cluster tissues; and (3) the presence of a leader peptide sequence [43]. Microcins usually exert antibacterial activity through their unusual, complex and complex mechanisms of action, called the Trojan horse strategy. For example, MccJ25 is an analog of an essential nutrient that is recognized by specific outer membrane receptors and further translocated to the periplasm [81]. The smallest antibacterial peptide of the microcin family was first isolated from the culture supernatant of an *E. coli* strain containing a 43 kb single-copy pMcC7 plasmid [82,83]. McC7 is an aspartate-tRNA synthetase (Asp-RS) inhibitor. After entering target bacteria, nonhydrolyzable inhibitors targeting Asp-RS can be generated to inhibit protein synthesis by target bacteria. The unique mechanism by which McC7 inhibits the emergence of bacterial resistance provides a promising way to develop novel antibacterial substances [79].

## 3. Structure of McC7

**Figure 1:**
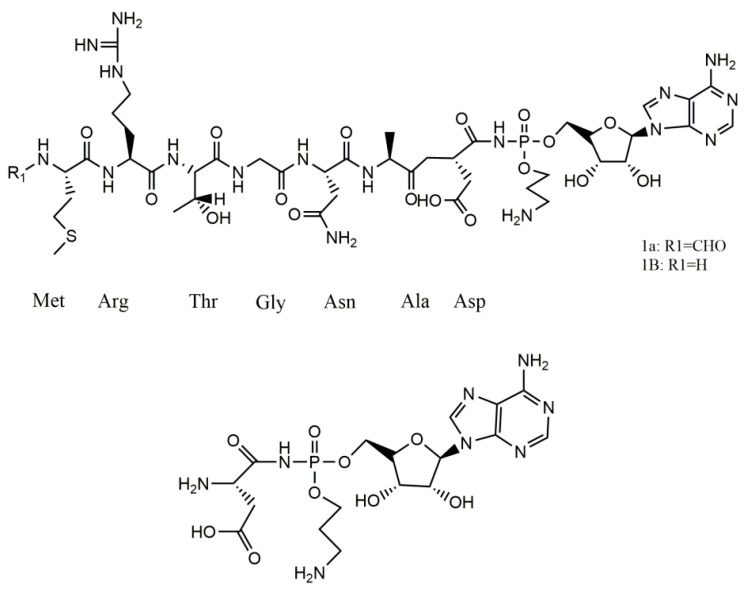
The structure of Microcin 7 (McC7). Intact McC7 (compound **1a**), the deformylated variant (compound **1b**), and processed McC7 (compound **2**) were obtained. (Reproduced with permission from [85]).

Understanding the structure of Microcin C7 (McC7) is essential for elucidating its antibacterial mechanisms. The structure of antimicrobial peptides (AMPs) is strongly related to their functions. As shown in Figure 1, complete McC7 (Compound **1a**) is composed of the MRTGNAD heptapeptide and modified adenosine monophosphate, which are linked to the α-carboxyl group of aspartic acid through an N-acyl phosphoramidite bond. The methionine at the N-terminus of the heptapeptide is formylated, and the phosphate group is modified by an aminopropyl group [40]. Once in sensitive cells, McC7 is treated by deformylase and several peptidases to hydrolyze the formyl group and peptide moiety at its N-terminus, releasing mature McC7 (Compound **2**). Mature McC7 targets Asp-RS in bacteria to exert its antibacterial effects [41,84].

## 4. Problems in the Application of AMPs and McC7 Biosynthesis

### 4.1. Problems in the Application of AMPs

The emergence and spread of drug resistance in both developed and developing countries necessitates the development of new antibacterial drugs to combat this natural phenomenon. Many antibiotics currently in use are derived from natural sources, such as low-molecular-weight secondary metabolites produced by microorganisms [1,2,3]. Bacteriocins possess advantages that make them good candidates for new alternatives to antibiotics. Considering that AMPs are peptides encoded by genes, genetic engineering is a feasible approach for their modification [33,34,35]. With the development of synthetic biology and metabolic engineering, the era of biosynthetic antimicrobial peptides has arrived. Ribosome-encoded AMPs, such as microcins, are a class of peptide molecules that have yet to become popular but have special prospects; for example, they can serve as clinical antibiotic substitutes. As these peptides are encoded by ribosomes, they can be obtained through genetic engineering methods.

At present, most AMPs can be synthesized chemically, but the synthesis yields are low, the purity is not high, and they are typically only used in laboratory research. It has been reported that the cost of chemically synthesizing 1 mg of AMP ranges from USD 100 to USD 1000. Therefore, the cost of chemically synthesizing large quantities of AMPs is exorbitant, and chemical synthesis methods are not suitable for the large-scale production needed for clinical research and commercial applications [86]. Recombinant expression is the most common method for the preparation of therapeutic AMPs and has been applied in many laboratories for the large-scale production of biosynthetic AMPs [87,88,89]. The recombinant expression of AMPs is more cost-effective than chemical synthesis. In addition, the process, development, and large-scale production steps have been optimized, and the existing equipment used for the production of therapeutic proteins is also suitable for the production of AMPs. However, the construction of recombinant expression systems and the large-scale expression, purity, stability, and safety of bioactive AMPs limit their clinical application and promotion. Therefore, the application of AMPs in clinical practice still faces many challenges: (1) the cost of AMPs synthesized by chemical methods is high, and the yields are low; (2) the construction of a natural AMP expression system is complicated, the yield of recombinant AMPs is low, isolation is difficult, and it is difficult to establish antibacterial and purification methods; (3) there are many types of AMPs, and although many antibacterial peptides have a broad spectrum, their antibacterial abilities vary greatly; (4) few AMPs have both antibacterial activity and immune regulation ability at the same time; (5) regarding safety issues associated with biosynthetic AMPs compared with antibiotics, biosynthetic AMPs that function in the body will be at higher concentrations than needed in vitro, and as a result, they are prone to producing cytotoxicity and high hemolytic activity, which limits their application in the body; and (6) the stability of AMPs, for example, the pH environment and various proteases and peptidases present in animal intestinal tracts, can inactivate most AMPs.

### 4.2. Biosynthesis of McC7

**Figure 2:**
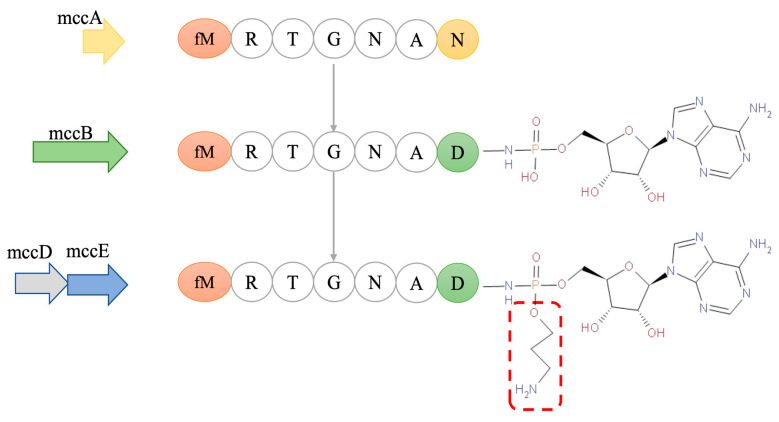
The McC7 structure formation process. The heptapeptide shown on the left is the product of *mccA*, which is adenylated by *mccB,* and the resulting peptide adenylate is decorated with an aminopropyl moiety in the reaction that requires *mccD* and *mccE*, resulting in mature McC7. (Reproduced with permission from [94]).

McC7 is produced by *E. coli* strains that carry the *mccABCDEF* gene cluster plasmid. The *mccABCDE* gene constitutes an operon that is produced under nutrient deficiency or cellular stress, while the *mccF* gene is transcribed independently [90]. The synthesis process for McC7 is shown in Figure 2, and the details are as follows: The peptide moiety (MRTGNAN) of McC7 is encoded by the *mccA* gene, which is a 21 bp gene and one of the shortest known protein-encoding bacterial genes [91]. The enzyme encoded by the *mccB* gene catalyzes the coupling of the heptapeptide to adenosine monophosphate and simultaneously converts the asparagine residue at the heptapeptide’s C-terminus to aspartic acid [92]. Under the action of the *mccD* and *mccE* genes, the aminopropyl group is linked to the catalytic product of the *mccB* gene through a phosphoamine bond with S-adenosylmethionine as the donor. The aminopropyl group is not essential for McC7, but the presence of an aminopropyl group increases the biological activity of McC7 by approximately 10-fold, possibly due to an increase in McC7′s affinity for its target enzyme. McC7 is exported from McC7-producing cells through the master facilitator superfamily efflux pump encoded by the *McC7* gene. The N-terminus of the heptapeptides of most McC7 produced in *E. coli* cells contains formylation modifications. However, reformylation and mature intermediates lacking aminopropyl groups are also produced to varying degrees [93].

In recent years, microcins produced by engineered bacteria have been used as potential drug treatment strategies for diagnosis and therapy. Sassone-Corsi et al. reported that microcins produced by *E. coli* Nissle 1917 could competitively expel pathogenic *Enterobacteriaceae* and relieve intestinal inflammation under specific environmental conditions [34]. Studies have also shown that microcin-producing *Enterobacteriaceae* can decrease the number of *Salmonella* and *E. coli* in the host intestine and intestinal inflammation in mice and poultry [34,95]. Studies by Palmer et al. have shown that MccH47 isolated from *E. coli* H47 could inhibit the growth of *Salmonella* and multidrug-resistant bacteria in vitro after translational modification [96]. At the same time, the research group further prepared a plasmid expression system of genetic probiotics and transferred this plasmid system to *E. coli* to demonstrate that this *E. coli* strain can produce microcins using the environmental signal of intestinal inflammation (sodium tetrasulfate) as the inducing molecule. This inhibited the inflammation caused by *Salmonella* [96,97]. Thus, it is necessary to establish a system for the high-efficiency expression of microcins by means of microbiology and molecular biology that does not rely on wild-type regulatory mechanisms.

## 5. Research Progress on McC7 Mutations

At present, research on the biomodification of microcins has two focuses: elucidating the positioning of the constant region and the variable region and identifying AA residues in active sites through mutagenesis studies to generate microcins with broad spectrum specificity, stability, and better derivatives with excellent antibacterial activities both in vivo and in vitro. With the development of site-directed mutagenesis and expression systems for structural genes, many lanthionine bacteriocin mutants have been designed and produced in vivo, improving the antibacterial activity of bacteriocins through bioengineering methods. The first nisin mutation was reported, and four years later, the activity of the T2S mutant increased [98,99]. Yuan et al. reported that several hinge region mutants exhibited antibacterial activity against gram-negative bacteria [100]. Rink et al. reported that I4K, S5F, and L6I, which were designed with a loop structure, exhibited improved antibacterial activity against some target strains [101]. A19C of the lantibiotic Pep5 introduces a novel thioether cross-linking bridge formed between Dhb at position 16 and Cys at position 19. This mutant has enhanced stability against hydrolysis by chymotrypsin [102].

Using the total chemical synthesis method, McC7 analogs with shorter peptide chain structures were synthesized. However, the associated experimental results showed that the antibacterial activity of analogs with shorter peptide chain structures was weakened due to the reduced membrane penetration efficiency of McC7 analogs. The peptide chain in mature McC7 is critical for the active uptake of YejABEF. Kazakov et al. were the first to apply site-directed mutagenesis to the *mccA* gene [103]. As reported, 2–7 codons for each *mccA* gene were systematically replaced with codons for 19 standard AAs, resulting in a total of 114 mutations. This method cannot be used to study the role of the first methionine of MccA peptides, as this residue is essential for translation initiation. The MccB enzyme can adenylate only the peptides ending in asparagine; therefore, the seventh codon of mccA cannot be mutated [85,104]. Finally, 28 mutants that did not significantly change the biological activities of the peptides were screened. Although the four mutants R2Y, R2M, A6M, and A6F lost their antibacterial activity, they had no effect on the maturation of McC7 according to mass spectrometry. Therefore, arginine at position 2 and alanine at position 6 may both serve as recognition molecules for cell uptake.

The effect of peptide length on McC7 trafficking in *E. coli* has been studied through chemical synthesis. The results showed that the McC7 peptide with AA shortening had significantly reduced biological activity, affecting yejABEF-mediated transport. Two necessary conditions for transport are a minimum peptide chain length of 6 AA and an N-terminal f-MR sequence [85]. Recombinant MccB protein has been used to prepare McC7 analogs with different peptide chain lengths. They found that the N-terminal extension of the MccA heptapeptide of *E. coli* did not affect the adenylation of MccB proteins [104]. The antibacterial activities of some of the McC7 analogs with N-terminal extensions increased. When the peptide chain length increased to 20 AA, both the yejABEF transporter and the SbmA transporter could transport the toxic part of McC7 into the target bacteria [105]. At the same time, the heptapeptide (MR) of McC7 only acts as a camouflage to transport the toxic part of McC7 into the target bacteria, and only when the toxic part of McC7 has entered the intracellular tissue of the target bacteria is it released to exert its antibacterial effect; this has almost become common practice in our society—an accepted fact [106]. More precisely, MR is only responsible for recognition of the yejABEF transporter on the target bacterial membrane, which is an indispensable part of the efficient transport of McC7 into target bacterial cells [84,93,107]. However, researchers have begun to wonder whether, in addition to MR’s ability to recognize and transport McC7, it might affect bacterial growth. Guijarro et al. showed that MR does not affect bacterial growth, even when MR is administered at doses up to 200 nM. In vitro, the inhibitory activity of the N-terminal formylated heptapeptide f-MRTGNAD and the precursor heptapeptide MRTGNA of MR were examined using a coupled translation transcription system. The results showed that 10 µM McC7 inhibited the incorporation of 35S Met into the newly synthesized MR protein. Additionally, the inhibition rate reached 50% within 30 min and 75% at 40 µM for 30 min. The results for f-MRTGNAD and McC7 were similar. The results of the *E. coli* growth experiment showed that 20 µM McC7 could prevent the growth of *E. coli*. However, even when these peptide concentrations reached 200 µM, they still had no effect on *E. coli* growth [40]. Notably, MC4100 cells were transformed with recombinant plasmids lacking any immune genes, and then, the growth of translucent colonies in liquid Luria–Bertani (LB) medium was examined. The results showed that the expression of the precursor heptapeptide MRTGNA could reduce the growth rate of bacteria, including bacteria in the stationary phase. Therefore, the authors concluded that the intracellular precursor heptapeptide MRTGNA inhibited bacterial growth [88]. Neither McC7′s f-MRTGNAD nor the heptapeptide MRTGNAD affect the growth of bacteria [41]. However, the experimental results revealed that f-MRTGNAD and the heptapeptide MRTGNAD of McC7 could inhibit protein synthesis, while the antibacterial results showed that f-MRTGNAD and the heptapeptide MRTGNAD had no effect on bacterial growth. Thus, the heptapeptide of McC7 is only responsible for the recognition of the inner membrane transporter YejABEF to transport McC7 to microbial cells, but it lacks the ability to inhibit microbial growth. This begs the following question: what is the significance of the McC7 Trojan horse antibacterial strategy?

Ran et al. demonstrated the effects of MR and two analogs—N-formylated heptapeptide (f-MR) and N-acetylated heptapeptide—on the growth of microbial cells [107]. Surprisingly, MR not only inhibited the activities of intracellular β-galactosidase, respiratory chain dehydrogenase, and 6-phosphogluconate dehydrogenase (6PGDH) but also inhibited the growth of *E. coli*. Eventually, at a lethal concentration of 5.34 mM for 10 min, cell death occurred. Changes in MR resulted in a slight increase in the lethal concentration. Cell membrane integrity at a lethal concentration confirmed that MR had an inhibitory effect but not through the destruction of cell membrane integrity. The novel properties of MR have shed light on McC7′s Trojan horse strategy and have opened new avenues for antibiotic design. According to the unique mechanisms by which McC7 exerts its effective and selective antibacterial activity, it can become a classifier for scientists to generate similar drugs through imitating these unique mechanisms and strategies, thus replacing the use of antibiotics in many fields. At present, a series of McC7 analogs are being developed to improve antibacterial activity and develop new antibiotics.

Yang et al. constructed trypsin-resistant mutants of the bacteriocin McC7 and studied their antibacterial activity [94]. They successfully constructed 19 mutants, including 14 mutants that had antibacterial activity against the *E. coli* Yej+ rimL strain: R2A, R2S, R2H, R2Y, R2L, R2I, R2V, R2M, R2T, R2G, R2F, R2Q, R2K, and R2D. The tolerance results showed that after trypsin, pepsin, and chymotrypsin treatment, the 12 mutants were stable and retained their antibacterial effects. The inhibition zone diameters (from largest to smallest) were R2Q, R2A, R2T, R2S, R2H, R2I, R2Y, R2V, R2M, R2G, R2D, and R2L [94]. However, after chymotrypsin treatment, the mutants were hydrolyzed. This phenomenon was similar to that reported by Yu et al. for MccJ25, which retained antibacterial activity even after enzymatic hydrolysis [12]. The authors continued to use the same method to construct mutants with substitution (T3P) or the addition of proline (RTP) at the second position after arginine. The results showed that neither mutant had antibacterial effects. The 12 mutants screened in the experiment were purified through high-performance liquid chromatography to obtain 12 pure mutants with purities greater than 80%. Mass spectrometry analysis was performed for each mutant, and the results showed that the molecular weight was in line with expectations. The *E. coli* Yej+ rimL strain was used as the indicator bacterium for determining the minimum inhibitory concentration. Compared with those of wild-type McC7, the MICs of the mutants were greater than those of wild-type McC7 (1.56 μg/mL), and the R2A, R2T, and R2Q values were significantly greater. The antibacterial ability of the three mutants outperformed that of the other 12 mutants, with MICs of 12.5, 25, and 25 μg/mL, respectively. This study led to the successful construction of 12 McC7 mutants that were resistant to trypsin and retained their antibacterial effects, established a purification method and provided a preliminary investigation of their antibacterial activity. The results of this study provide basic scientific data on the use of trypsin-resistant McC7 mutants as drug candidates for therapeutic antibiotics [94].

## 6. McC7′s Antibacterial Mechanism

**Figure 3:**
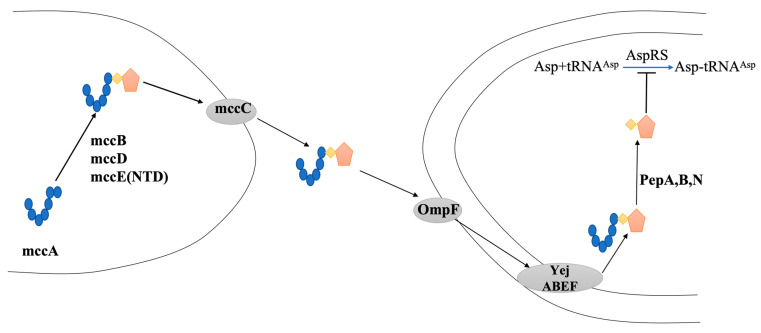
The McC7 structure formation process. The heptapeptide shown on the left is the product of mccA, which is adenylated by mccB, and the resulting peptide adenylate is decorated with an aminopropyl moiety in the reaction that requires mccD and mccE, resulting in mature McC7. (Reproduced with permission from [94]).

The mode of action of mature McC7 is shown in Figure 3. When all translation modifications are complete, mature McC7 first passes through the pore protein OmpF on the outer membrane of the bacterial cell and then through the inner membrane of the bacterial cell. The ABC transporter YejABEF recognizes f-Met at the N-terminus of the McC7 peptide and transports McC7 into cells; once it is inside a target bacterial cell, McC7 processing begins [108]. First, the formyl group at the N-terminus is removed under the action of deformylases; next, the N-terminal methionine is removed by one or more of the three aminopeptidases (PepA, PepB, and PepN). When the peptide’s 6th and 7th residue peptide bonds are hydrolyzed, the nonhydrolyzable analog of aspartyl adenosine is released [84]. The tRNA aminoacylation reaction catalyzed by aminoacyl-tRNA synthetase includes two steps. The first step is the recognition of the amino acid catalyzed by aminoacyl-tRNA synthetase and the substrate adenosine triphosphate (ATP). Additionally, under the catalysis of aminoacyl-tRNA synthetase, the carboxyl group of the amino acid and the phosphate on AMP can form an ester bond. Furthermore, a molecule of PPi is released; in the second step, the amino acid is linked to ribose at the 3′ end of tRNA through the formation of an ester bond. Processed McC7 is a nonhydrolyzable analog of aspartyl adenosine monophosphate that binds to Asp and ATP; that is, it inhibits the first step in translation, thereby inhibiting protein synthesis [106].

### 6.1. The Immune Mechanism

When seeking to improve the antibacterial potential of bacteriocins, a crucial problem is how to limit their cytotoxicity to host cells. For example, in the *MccJ25* gene cluster, mcjD overexpression resulted in toxicity. McC7 has autoimmune genes, and its autoimmunity involves two proteins (MccC and MccE). When McC7 reaches a certain concentration in cells, McC7 acts as an efflux pump and can expel McC7 in cells to slow the accumulation of toxic substances. MccE detoxifies McC7 through acetylation, and acetylated McC7 is subsequently converted into nontoxic compounds that cannot inhibit aspartyl tRNA synthetase [109,110]. MccC is considered a natural substrate of RimL. Therefore, RimL—one of the Rim proteins of *E. coli*—helps to increase McC7 resistance [110].

Another immune function of host cells against accumulated McC7 is mediated by the MccF protein. MccF detoxifies intact and processed McC7 by cleaving the amide bond linking the peptide moiety and the nucleotide moiety of McC7 [111]. Aspartame sulfonamide adenosine acid (DSA) is an analog of processed McC7. It can be cleaved to release DSA, indicating that MccF does not require the McC7 peptide for cleavage [112]. MccF is more specific than MccE acetyltransferase because MccF can recognize aspartate and glutamate but not leucine. MccE acetylates all aminoacylsulfonyl adenylate, except for prolylsulfonyl adenylate nucleic acid [109]. Therefore, McC7 host cells undergo self-immunity under the joint action of the MccC export pump, MccE acetyltransferase, and MccF protease.

### 6.2. The Immune Mechanism

When McC7 enters target cells, deformylase and aminopeptidase remove the hexapeptide responsible for transporting McC7 and release the toxic moiety that does not possess membrane-penetrating properties, thus inhibiting the growth of bacteria [108]. McC7 belongs to the Trojan horse series of antibacterial peptides. As it is usually difficult for aminoacyladenosine to penetrate bacterial cells, processed McC7 mimics the aminoacyladenosine intermediate and adopts the “Trojan horse” strategy to bind the active ingredient to specific modules or signal peptides to overcome the risk of aa (where ‘aa’ represents the amino acid in the single-letter code). The problem is that AMPs and their analogs are difficult for cells to absorb [37]. This unique mode of action of McC7 makes it an attractive model for the design of aminoacyl-tRNA synthetase inhibitors rather than cloned analogs of natural McC7, which inhibits aspartyl of tRNA synthetase [113]. Saturation mutagenesis was performed at the molecular level toward the last position of the heptapeptide, but the synthesis was unsuccessful. The AA at the seventh position could be changed through total chemical synthesis, and a total of three McC7-like compounds were obtained, including the termini of aspartate, glutamate, or leucine, which are linked to adenosine through a nonhydrolyzable sulfamate bond. Although these analogs lack the N-terminal formyl group, they are still active and bind to wild-type McC7, have the same mechanism of action, and target aspartyl tRNA synthetase, glutamyl tRNA synthetase, and leucyl tRNA synthetase. At present, the most commonly reported way for McC7 to exert its antibacterial effects is through its targeting of bacterial cells. Therefore, it is crucial for scientific researchers to understand the antibacterial mechanism by which McC7 enters bacterial cells to exert antibacterial effects [113]. However, whether McC7 has an outer membrane mechanism or multiple intracellular targets remains unclear. Bacteria may exert a variety of independent mechanisms of action. Whether the different mechanisms are connected or combined to better inhibit and kill the target bacteria also needs to be explored.

## 7. In Vivo Function and McC7 Application

### 7.1. In Vivo Anti-Infection Studies: Direct Antibacterial and Anti-Inflammatory Activities and Intestinal Health Regulation

Pathogenic *E. coli* and *Salmonella* are the most common enteric pathogens that not only affect human and animal health but also cause food poisoning and food-related epidemics worldwide [114,115,116]. Typically, pathogenic bacterial infections and their harmful effects are prevented and treated with antibiotics. However, the overuse of antibiotics has led to increasing bacterial resistance and an increased treatment failure rate for various infectious diseases. Currently, the emergence of drug resistance is one of the most challenging global health crises [117,118,119]. Moreover, the pace of new drug development in the 21st century has been slow. Advanced methods, including genomics, high-tech chemical synthesis, and high-throughput screening, have been unsuccessful in promoting the discovery of new antibiotics, especially new antibiotics for the treatment of gram-negative Enterobacteria [120]. Therefore, new strategies to combat antibiotic resistance are increasingly necessary. Accordingly, there is an urgent need to promote the search for more effective and less toxic antibacterial drugs for the treatment of bacterial infections.

**Figure 4:**
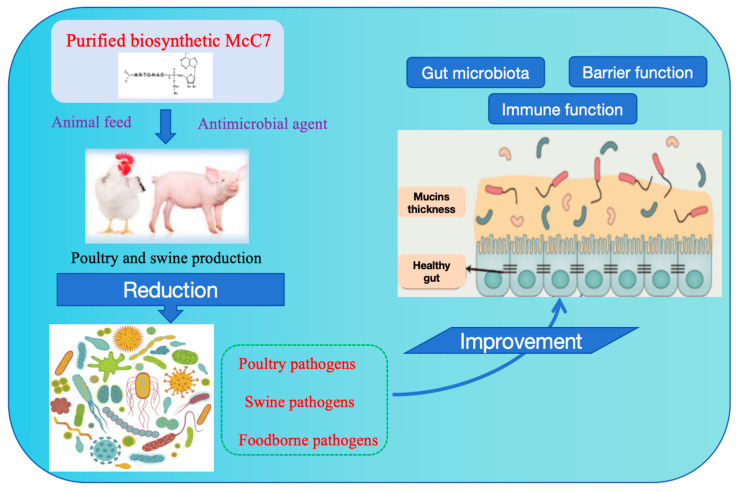
The beneficial impacts of McC7 on intestinal health. McC7 is a potential antibacterial–immunomodulatory peptide that can improve growth performance and decrease diarrhea by regulating the gut microbiota, alleviating intestinal inflammatory responses, and enhancing intestinal barrier function in weaned pigs and poultry birds.

Due to its strong antibacterial activity and unique antibacterial mechanism, natural McC7 has attracted attention as a potential antibacterial agent for humans, animals, and food. Most previous studies on McC7 have focused on the associated biochemical and antibacterial mechanisms and structural characteristics [33,85]. Based on the antibacterial activity and distinctive mode of action of McC7, it could be a promising class of antibacterial compound that has potential application prospects in animal nutrition as a growth promoter, antibacterial agent, and immunostimulant. These prospects are reviewed in this study. As subtherapeutic doses of antibiotics are critical for improving growth and feed conversion efficiency, the use of in-feed antibiotics has become a common practice in animal feed production by reducing microorganism activity in the gastrointestinal tract. However, the continued use of antibiotics as growth promoters is related to the development, spread, and proliferation of antibiotic resistance produced by resistant microorganisms throughout the food chain. This has elevated consumers’ awareness of potential negative health effects and environmental issues. Therefore, since the ban on the use of antibiotics as feed additives in the European Union in 2006 and the comprehensive reduction in the use of antibiotics worldwide, new methods have emerged to promote growth in production animals. McC7 has been established as a promising antimicrobial compound with potential applications in the animal production sector. Its beneficial aspects (Figure 4) will be reviewed in the following sections.

### 7.2. Applications in Poultry and Pigs Production

As a feed additive, McC7 is an ideal solution for traditional or antibiotic-free livestock production systems. In a large group-feeding experiment involving 300 healthy 1-day-old AA male chicks, Dai et al. reported that dietary McC7 supplementation can improve the growth performance of broilers, enhance their immune function, improve the morphology and structure of the intestinal tract, enhance intestinal barrier function, and regulate intestinal microbes. This study indicated that McC7 may be a promising substitute for traditional antibiotics. Importantly, the effects varied by dose. For example, 4 mg/kg McC7 had the greatest effect on the feed/gain (F/G) ratio and the expression of tight junction proteins, while 6 mg/kg McC7 had the greatest effects on boosting immunity and anti-inflammatory function and maintaining intestinal environment homeostasis in broilers. This study also showed that the effect of McC7 was greater than that of antibiotics, and this effect was more obvious in the later period of broiler rearing. This may have occurred for two reasons: first, the effect of McC7 is cumulative, and second, broiler chickens consumed more McC7 in the later stages of feeding [121].

Based on these study results, we inferred that McC7 has an overall mode of action in broiler chickens: McC7 has antibacterial activity against gram-negative pathogens in the intestinal tract, resists pathogen invasion, and regulates the intestinal microbiota. Moreover, as an immunomodulator, McC7 can also regulate intestinal immune function, maintain intestinal microbiota balance, and promote intestinal health. As the gut is the largest immune and absorptive organ in the body, McC7 can further boost production by promoting intestinal health and simultaneously improving serum indicators or improving intestinal morphology through antioxidant and anti-inflammatory activities.

The prevention of bacterial diarrhea in piglets, control of the incidence of intestinal inflammatory disease, improvement of production performance, and reduction of pig mortality are the core tasks of pig production at present and presumably will remain so long into the future [8,11,15]. Therefore, it is urgent to develop efficient, safe, and green antibiotic substitutes and to study their mechanisms of action to address the core scientific issues associated with the use of antibiotic substitutes, thus ensuring the efficient, healthy breeding and sustainable development of piglets. The positive effects of McC7 on the gut of weaned piglets are shown in Figure 4. Shang et al. investigated the effects of dietary supplementation with high doses of McC7 on gut health in weaned piglets. At each stage of the experiment, there were no significant differences in the average daily gain (ADG), average daily feed intake, or F:G between the 5000 mg/kg McC7 dietary supplementation group and the control group. However, the ADG at 0–14 d was significantly lower than that in the control group. In the dietary supplementation with 500 mg/kg McC7 treatment group, the F:G ratios in the experimental stages were significantly greater than those in the dietary supplementation with 500 mg/kg McC7 treatment group. Additionally, the routine blood test and organ index results presented no significant differences. Additionally, dietary supplementation with McC7 at 0–5000 mg/kg had no significant effect on the tissue morphology of the duodenum, jejunum, ileum, liver, or kidneys of weaned pigs. Under the conditions of this experiment, the suitable amount of dietary adenosine heptapeptide added was 500 mg/kg, which is a 10-fold increase in safety [122].

Next, the authors studied the effects of varying levels of dietary McC7 supplementation on growth performance, the diarrhea rate, nutrient digestibility, immune performance, intestinal morphology, the intestinal barrier, and faecal microbes in weaned piglets. Shang et al. reported that dietary supplementation with 500 mg/kg McC7 could increase ADG throughout the whole period (0–28 d) and reduce F:G in the late period (15–28 d) and throughout the whole period, which improved weaning efficiency. Regarding the production performance of piglets from 0 to 28 d, the diarrhea frequency and the diarrhea index in the groups supplemented with 250, 500, and 750 mg/kg McC7 were significantly lower than those in the control group; the 500 mg/kg McC7 group presented the lowest values, and diarrhea was relieved in all treatment groups; dietary supplementation with 500 mg/kg McC7 improved the digestibility of crude fat and calcium in weaned pigs; dietary supplementation with 250 mg/kg McC7 caused IgG levels in the McC7 group to be significantly greater than those in the control group at 14 d and 28 d; and tumour necrosis factor α levels in the diets supplemented with 250 and 500 mg/kg McC7 at 28 d were significantly lower than those in the control group. The expression level of interleukin-10 in the ileum was significantly greater in the 500 and 1000 mg/kg McC7 treatment groups than in the control group. Dietary supplementation with 500 or 1000 mg/kg McC7 improved the intestinal morphology of the duodenum and ileum of weaned piglets, facilitating digestion and absorption. Furthermore, McC7 treatment markedly improved epithelial barrier function by enhancing tight junction protein expression and the gut microbiota composition by increasing the beneficial bacterial composition, suggesting that McC7 treatment is helpful for improving gut health and intestinal villus development in weaned piglets [122].

### 7.3. Applications in Immunomodulation and Infection Prevention

Boosting the innate immunity of the host intestine is a key and feasible way to solve the global problem of bacterial resistance, thus reducing the use of antibiotics [123,124,125,126]. Antibacterial peptides not only inhibit and kill pathogenic microorganisms but also have important immunoregulatory functions and have been recognized as substitutes for antibiotics with high application potential [25,26,27,28]. Dai et al., used a cyclophosphamide-induced immunosuppressed mouse model to explore the effects of the antibacterial peptide McC7 on immunity and intestinal barrier function in mice and elucidated the underlying mechanism involved. The results showed that McC7 relieved weight loss in mice caused by cyclophosphamide and relieved the inhibitory effect of cyclophosphamide on the proliferation of lymphocytes in the spleen and the phagocytosis of peritoneal macrophages in mice in a dose-dependent manner. McC7 significantly reduced the serum D-lactate and diamine oxidase levels and increased the expression of the tight junction proteins ZO-1, Claudin-1, and Occludin and the expression of mucin 1, mucin 2, and secretory immunoglobulin A (sIgA) in the jejunum and colon. McC7 significantly increased the height of the villi and the ratio of villous crypts in the jejunum, decreased the crypt depths in the jejunum and colon, and improved the morphology of the jejunum and colon. In addition, McC7 significantly increased the abundance of *Lactobacillus* and Bifidobacterium while decreasing the abundance of *E. coli* in the colon of mice [127].

**Figure 5:**
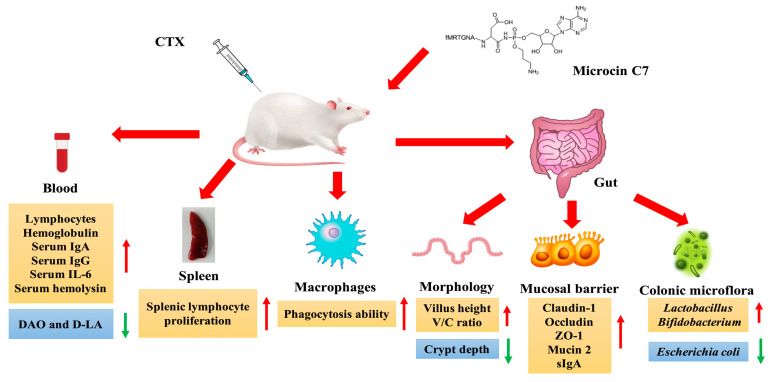
McC7 exhibits protective and immunomodulatory functions in a CTX-induced immunodeficient mouse model. McC7 can improve clinical symptoms, enhance the phagocytosis of immunized peritoneal macrophages, enhance epithelial barrier function, improve intestinal microbiota composition, and inhibit proinflammatory responses after CTX treatment. McC7, a viable immunomodulator peptide, can be a potential solution for treating pathogenic microbial infection in the postantibiotic era and can be used in animal feed, functional foods, and immunological regimens.

As shown in Figure 5, McC7 not only has a direct antibacterial effect but can also activate immune function and improve intestinal health. Therefore, the AMP McC7 has potential as a replacement for antibiotics. Various mechanisms underlying the improvement of animal immunity by McC7 have been hypothesized, such as the following: (1) adaptive immunity is boosted through increasing the splenic lymphocyte proliferation rate; (2) the innate immunity of animals is improved through stimulating the phagocytic activity of macrophages; and (3) reducing intestinal permeability, improving intestinal morphology, increasing the expression of intestinal tight junction proteins (e.g., Caludin-1, ZO-1, and Occludin) and regulating mucin 1, mucin 2 and sIgA in the jejunum and colon. The colon microbiota boosts intestinal mucosal immunity in animals. All of these results indicate that McC7 is an ideal immunomodulator and can be used as a feed additive to improve immune function in animals.

Using animals as a model under uninfected and pathological infection conditions, the study of McC7 in animal husbandry, food, or clinical applications plays a role in the promotion and application of McC7 as a sustainable, safe, and effective antibiotic substitute in resistance-free diets for weaned piglets. Additionally, activating intestinal factors and increasing their expression with dietary McC7 represent new targeted methods for preventing and treating inflammatory bowel disease and reducing mortality.

## 8. A Potential Method for Improving the Antibacterial Spectrum of McC7 via the Conjugation of Micro/Nanoparticles

Chitosan nanoparticles (CNs) have become potential candidates for use as antibiotic substitutes due to their broad-spectrum antibacterial activity, antioxidant activity, and low toxicity both in vivo and in vitro [19,128]. In addition, CNs can act as drug delivery carriers or can be coupled with other protein molecules to exert antibacterial effects. For example, after egg yolk antibody (IgY) was used to couple CNs, CN-IgY had strong specific antibacterial activity without inhibiting beneficial bacteria, selectively removing pathogenic microorganisms from the gastrointestinal tract [20]. The above work suggested that CNs can serve as carriers to improve the biological efficiency of other substances and can also act as antibacterial agents to exert anti-infective effects and relieve intestinal inflammation caused by pathogenic microbial infections. Yu et al. successfully prepared an antibacterial polymer nanoparticle using the antibacterial peptides MccJ25 and CNs, named CNMs, and confirmed that the CNMs have high antibacterial activity, good stability, high safety, and do not induce bacterial mutations or drug resistance [23]. McC7 and MccJ25 belong to the same bacteriocin family. Based on its structure and function, we can try to expand the antibacterial spectrum of McC7 and improve its antibacterial activity based on the use of chitosan and nano/microchitosan particles.

In recent years, reports have shown that polymeric nanoparticles based on AMPs have broad application potential as antibacterial agents due to their strong antibacterial properties. Liu et al. reported that a series of nanoparticles self-assembled from an amphipathic peptide (CG3R6TAT) had excellent bactericidal ability and significantly reduced the colonization of *Staphylococcus aureus* in the intestines of mice [16]. Lam et al. developed peptide polymer nanoparticles composed of lysine and valine residues that not only had good antibacterial activity against gram-negative bacteria but also, more importantly, could kill drug-resistant *E. coli* and multidrug-resistant pathogens [17]. Its antibacterial mechanisms are multifaceted and include destruction of the integrity of the bacterial outer membrane and the cytoplasmic membrane. This also provides a candidate method for improving the antibacterial activity of McC7.

## 9. Conclusions and Prospects

In the global postantibiotic era, efforts have been undertaken to curb antibiotic resistance, with some success. Unfortunately, in various fields, such as food, clinical medicine, and animal husbandry, antibiotic resistance is developing at a speed that exceeds the speed of the discovery of new antibiotics. Under these circumstances, several issues, such as the decline in animal resistance to inflammation and disease, the decrease in production efficiency, and the increase in production costs, have become prominent, thus detracting from China’s livestock industry.

Improving the host’s intestinal innate immunity and inhibiting the growth of pathogenic microorganisms are crucial and feasible solutions to the global problem of bacterial resistance and serve as a potential means for reducing antibiotic use. McC7 has attracted significant attention due to its excellent bioactivity and thus has broad application potential in a wide range of food, animal feed, and medical contexts. As a bactericidal or immunomodulatory agent for the direct treatment of bacterial infections, McC7 has great potential for treating bacterial infections with strong antibiotic resistance. Although McC7 has enormous advantages and potential as a novel antibacterial agent or immune regulator, there is still much work to do. For example, there are scarce available in vitro and in vivo data regarding its long-term safety, toxicity, and drug resistance. In future studies, the effects of Mcc7 can be studied in ruminants. In any case, we hope that this review will help readers understand the immense potential of McC7 in terms of its ability to promote growth in different animals, including ruminants, as an antibiotic substitute and as a means for the treatment of drug-resistant bacteria.

## Figures and Tables

**Table 1:** Principle classification, structure, and size of microcins.

Microcins	Class	Precursor	Leader Peptide	Mature Microcin	MW, Da	Featured	Bibliography
		Number of AA	Number of AA	Number of AA			
MccB17	I	69	26	43	3093	Lasso structure, heterocycle containing or others	[44,77]
McC7/51	I	7	0	7	1177		
MccJ25	I	58	37	21	2107		
MccV	IIa	103	15	88	8733	Unmodified bacteriocin, linear, non-pediocin-like	
MccL	IIa	105	15	90	8884		
Mcc24	IIa	90	17	73	7475		
MccE92	IIb	103	19	84	8717	Colicin V-like bacteriocin, and Siderophore-microcin family	
MccM	IIb	92	15	77	7886		
MccH47	IIb	75	15	60	7283		
MccI47	IIb	77	15	62	4865		
